# Chronic Administration of *Calendula officinalis* Ethanolic Extract Mitigates Anxiety-like Behavior and Cognitive Impairment Induced by Acute Scopolamine Exposure in Zebrafish

**DOI:** 10.3390/ph18101483

**Published:** 2025-10-02

**Authors:** Lucia-Florina Popovici, Ion Brinza, Simona Oancea, Lucian Hritcu

**Affiliations:** 1 Department of Agricultural Sciences and Food Engineering, “Lucian Blaga” University of Sibiu, 7–9 Ion Ratiu Street, 550024 Sibiu, Romania; luciaflorina.popovici@ulbsibiu.ro; 2 Faculty of Sciences, “Lucian Blaga” University of Sibiu, 5–7 Ion Ratiu Street, 550024 Sibiu, Romania; ion.brinza@ulbsibiu.ro; 3 Department of Biology, Faculty of Biology, Alexandru Ioan Cuza University of Iasi, 700506 Iasi, Romania; hritcu@uaic.ro

**Keywords:** *Calendula officinalis*, zebrafish, scopolamine, anxiety-like behavior, cognitive performance, oxidative stress, acetylcholinesterase

## Abstract

**Background/Objectives:** Scopolamine (SCO) is widely employed as a pharmacological model of anxiety and amnesia in both rodents and zebrafish, the latter representing a valuable translational model in neuropsychopharmacology. The present study aimed to evaluate the neuroprotective and antioxidant potential of chronic administration of an ethanolic extract from *Calendula officinalis* flowers (CEE). **Methods:** Adult zebrafish (n = 10/group, both sexes) were exposed to CEE at concentrations of 1, 3, and 10 mg/L, administered daily for 22 consecutive days. After the initial 7-day pretreatment period, fish were challenged with SCO (100 μM, immersion for 30 min) followed by behavioral testing, including the Novel Tank Diving Test, Light/Dark Test, Novel Approach Test, Y-Maze, and Novel Object Recognition. Subsequently, brain homogenates were analyzed for acetylcholinesterase (AChE) activity, antioxidant enzymes (superoxide dismutase—SOD, catalase—CAT, glutathione peroxidase—GPx), reduced glutathione (GSH), protein carbonyls, and malondialdehyde (MDA). **Results:** Chronic CEE administration significantly attenuated scopolamine-induced anxiety-like behaviors and improved spatial memory (Y-maze) and recognition memory (NOR), as well as reduced anxiety-like behavior in the SCO-induced zebrafish model. Biochemical analyses revealed that CEE restored AChE activity, enhanced the activity of SOD, CAT, and GPx, and increased GSH levels, while concomitantly reducing protein oxidation and lipid peroxidation. The most pronounced effects were observed at 3 mg/L, which nearly normalized both behavioral and biochemical parameters. **Conclusions:** The CEE exerted anxiolytic and procognitive effects in zebrafish through combined cholinergic and antioxidant mechanisms. These findings highlight its translational potential as a promising candidate for the prevention and treatment of anxiety-related and cognitive disorders.

## 1. Introduction

Scopolamine (SCO) is a tropane alkaloid with non-selective antimuscarinic activity (M_1_–M_5_), clinically used in the prevention of motion sickness and postoperative nausea, but widely applied in preclinical research as a pharmacological model for the transient induction of cholinergic dysfunction and cognitive deficits [1,2]. Due to its ability to cross the blood–brain barrier (BBB) and to block central muscarinic receptors, SCO induces behavioral and cognitive alterations characteristic of cholinergic hypofunction, manifested as impairments in attention, working, and episodic memory, as well as anxiety or sedation, depending on dose and species [3,4]. In humans, a recent meta-analysis highlighted robust cognitive deterioration, particularly following parenteral administration, thereby supporting the translational validity of this “memory loss” model [5,6]. From a pharmacological perspective, SCO differs from other cholinergic antagonists such as biperiden, as it more faithfully reproduces advanced stages of cognitive decline typical of Alzheimer’s disease (AD), whereas biperiden is more closely related to early cholinergic impairment observed in normal aging or mild cognitive impairment (MCI) [3]. Beyond its cholinergic effects, preclinical studies have demonstrated that SCO induces oxidative stress and neuroinflammation by reducing the levels of antioxidant enzymes, increasing lipid peroxidation (MDA), and activating the NLRP3 inflammasome, a molecular pathway relevant in AD pathogenesis [7]. SCO is widely used as an experimental model for the induction of anxiety and memory deficits both in rodents [8] and in zebrafish (*Danio rerio*) [9], a species that has emerged as a valuable translational model in neuropsychopharmacology.

In recent research, zebrafish have proven to be an efficient translational model for investigating these processes, due to their sensitivity to cholinergic perturbations, well-characterized exploratory behavior, and validated pharmacological responses. In this species, SCO effects are dose-dependent and context-specific: moderate doses (100–200 μM) impair recognition memory, fear learning, and *c-fos* gene expression in the telencephalon, whereas higher doses (≈800 μM) may paradoxically induce anxiolytic effects. Moreover, aversive memory impairment may persist for up to seven days, highlighting the model’s sensitivity to medium-term retention assessments [9,10]. Thus, SCO represents a validated pharmacological model for controlled induction of cholinergic dysfunction, oxidative stress, and cognitive deficits, providing a robust experimental framework for testing novel procognitive, anxiolytic, or neuroprotective agents.

*Calendula officinalis* (marigold, Asteraceae) has been traditionally employed for its anti-inflammatory, antioxidant, and wound-healing properties, being rich in flavonoids (quercetin, apigenin, isorhamnetin), triterpenoids (faradiol, ursolic/oleanolic acids), carotenoids, and phenolic acids; in addition, marigold is also classified as an edible flower with a favorable biocompatibility profile, supporting its biomedical relevance [11,12,13,14]. However, its bioactivity is highly dependent on the extraction method/solvent (including 70% ethanol), which critically impacts phenolic load and antioxidant capacity—hence the importance of rigorous extract reporting [15]. Recent enzymology and docking studies indicate that *Calendula*-derived compounds may inhibit target enzymes (e.g., carbonic anhydrase IX; inhibitory effects also reported on cholinesterases in other preparations), thereby supporting a potential enzymatic modulation relevant for the cholinergic–redox axis [16].

Although there is solid evidence for the antioxidant and anti-inflammatory effects of *C. officinalis*, studies in adult zebrafish assessing the impact of an ethanolic flower extract on anxiety and cognitive function in the cholinergic SCO model are lacking. Based on these considerations, the present study aimed to investigate whether chronic administration of *C. officinalis* ethanolic extract (CEE; 1, 3, 10 mg/L) could attenuate anxiety-like behavior, improve cognitive performance, and normalize oxidative status in zebrafish exposed to SCO.

## 2. Results

### 2.1. Effects of CEE on Anxiety-like Behavior in Zebrafish

#### 2.1.1. Novel Tank Diving Test (NTT)

**Figure 1:**
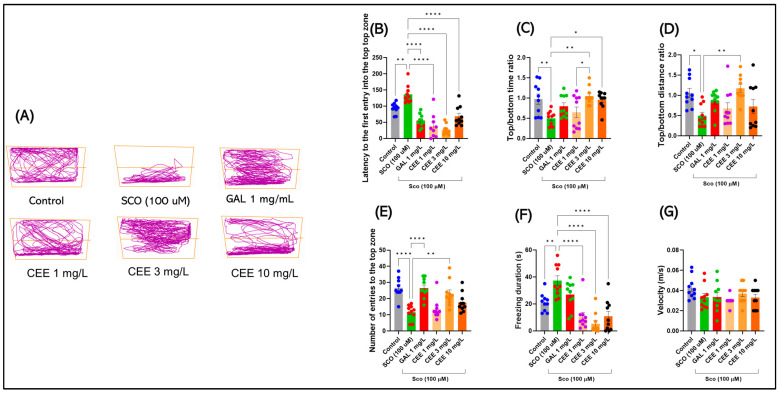
Effects of scopolamine (SCO) and *Calendula officinalis* ethanolic extract (CEE) in the Novel Tank Diving Test (NTT). (**A**) Representative track plots for each group: Control, SCO (100 μM), Galantamine (GAL, 1 mg/L), and CEE 1–10 mg/L. (**B**–**G**) Quantified parameters: (**B**) Latency to the first entry into the top zone; (**C**) Top/bottom time ratio; (**D**) Top/bottom distance ratio; (**E**) Number of entries to the top zone; (**F**) Freezing duration; (**G**) Mean swimming speed (velocity). Data are expressed as mean ± SEM (n = 10 per group). * *p* < 0.05, ** *p* < 0.01, and **** *p* < 0.0001.

Representative track plots (Figure 1A) illustrate the marked differences in vertical exploration between groups. Control fish displayed a balanced distribution of swimming across both zones, while SCO-exposed animals predominantly remained at the bottom, exhibiting reduced transitions to the upper zone and longer immobility periods. In contrast, zebrafish treated with CEE, particularly at 3 mg/L, showed restored exploration of the upper zone and decreased freezing, with trajectories comparable to or exceeding those of controls. Galantamine (GAL) treatment produced a similar anxiolytic profile, further validating the pharmacological sensitivity of the model.

Acute SCO administration induced a clear anxiogenic profile in zebrafish. Compared with the control group, SCO-exposed fish exhibited a significantly increased latency to the first entry into the upper zone (F(5,54 = 24.12, *p* < 0.0001) (Figure 1A), a reduced number of entries (F(5,54) = 11.22, *p* < 0.0001) (Figure 1E), and decreased time (Figure 1C) and distance ratios (Figure 1D) in the top/bottom zones (F(5,54) = 5.215, *p* = 0.0006 and F(5,54) = 4.411, *p* = 0.0019, respectively). In parallel, the duration of freezing behavior was markedly increased (F(5,54) = 14.81, *p* < 0.0001) (Figure 1F), further confirming the exacerbation of anxiety. By contrast, mean swimming speed did not differ significantly between groups (F(5,54) = 1.66, *p* = 0.160) (Figure 1G), suggesting that SCO specifically affected anxiety-related parameters without altering baseline locomotor activity.

Chronic treatment with CEE counteracted these effects in a dose-dependent manner. The 3 mg/L dose most effectively restored vertical exploration behaviors, reducing latency and freezing to levels comparable to or even lower than those of controls (**** *p* < 0.0001 vs. SCO; ** *p* = 0.0091 vs. Control for freezing) (Figure 1B,F). Administration of 1 mg/L produced moderate improvements, whereas 10 mg/L exerted intermediate effects, still maintaining a significant reduction in freezing compared with SCO. GAL induced a robust anxiolytic effect, comparable to that observed with CEE at 3 mg/L, thereby confirming the pharmacological validity of the model employed.

#### 2.1.2. Light/Dark Test (LDT)

**Figure 2:**
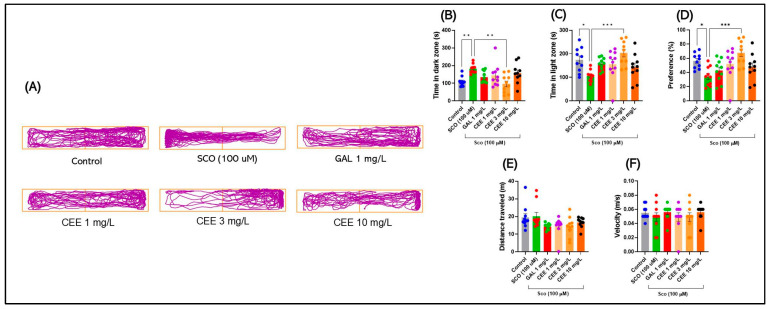
Effects of scopolamine (SCO) and *Calendula officinalis* ethanolic extract (CEE) in the Light/Dark Test (LDT). (**A**) Representative track plots for each group: Control, SCO (100 μM), Galantamine (GAL, 1 mg/L), and CEE at 1, 3, and 10 mg/L. (**B**–**F**) Quantified parameters: (**B**) Time spent in the dark zone; (**C**) Time spent in the light zone; (**D**) Preference index for the light zone; (**E**) Total distance traveled; (**F**) Mean swimming speed (Velocity). Data are expressed as mean ± SEM (n = 10 per group). * *p* < 0.05, ** *p* < 0.01, and *** *p* < 0.001.

Representative track plots (Figure 2A) clearly illustrate the contrast between the behavior of zebrafish exposed to SCO, which displayed reduced mobility and a strong preference for the dark compartment, and that of animals treated with CEE or GAL, which more frequently explored the illuminated compartment in the LDT.

Acute SCO exposure significantly increased the time spent in the dark compartment (F(5,54) = 4.717, *p* = 0.0012) and correspondingly reduced the exploration time in the light compartment (F(5,54) = 4.521, *p* = 0.0016), compared with controls (*p* < 0.05; Figure 2B,C). SCO also decreased the preference index for the light zone (F(5,54) = 4.886, *p* = 0.0009), indicating a robust anxiogenic profile (Figure 2D). CEE treatment counteracted these effects in a dose-dependent manner. Chronic administration of CEE at 3 mg/L significantly reduced the time spent in the dark zone and increased the preference for the light compartment compared with SCO (*p* < 0.01), an effect comparable to that of GAL (Figure 2B–D). The 10 mg/L dose produced intermediate effects, although without statistical significance in all cases.

Regarding locomotor parameters, the total distance traveled showed only a moderate trend toward differences between groups (F(5,54) = 2.55, *p* = 0.038), whereas swimming speed was not significantly affected by any treatment (F(5,54) = 0.568, *p* = 0.724) (Figure 2E,F). These findings confirm that the anxiolytic effects of CEE cannot be attributed to changes in global locomotor activity.

#### 2.1.3. Novel Approach Test (NAT)

**Figure 3:**
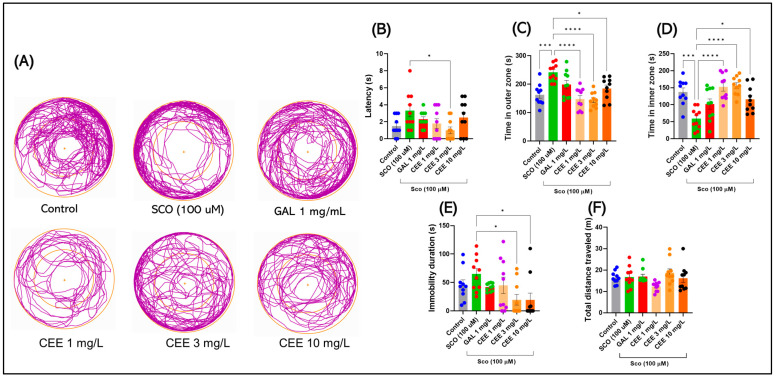
Effects of scopolamine (SCO) and *Calendula officinalis* ethanolic extract (CEE) in the Novel Approach Test (NAT). (**A**) Representative track plots for each group: Control, SCO (100 μM), Galantamine (GAL, 1 mg/L), and CEE 1–10 mg/L. (**B**–**F**) Quantified parameters: (**B**) Latency to the first entry into the central zone; (**C**) Time spent in the outer zone; (**D**) Time spent in the inner zone; (**E**) Immobility duration; (**F**) Total distance traveled. Data are expressed as mean ± SEM (n = 10 per group). * *p* < 0.05, *** *p* < 0.001, and **** *p* < 0.0001.

Representative track plots in Figure 3A reveal clear group differences in spatial exploration. Compared with controls, scopolamine-exposed fish spent significantly less time in the central zone and displayed a marked preference for the periphery, consistent with an anxiogenic profile. In contrast, treatment with CEE, particularly at 3 mg/L, restored exploratory behavior toward the center and reduced edge-dwelling, with a pattern comparable to that observed in galantamine-treated fish. Lower doses (1 mg/L) produced moderate effects, while 10 mg/L showed intermediate outcomes, still promoting a more balanced exploration than SCO alone.

Acute SCO administration induced a clear anxiogenic profile in zebrafish in the NAT. Compared with the control group, SCO-exposed fish showed a significant reduction in the time spent in the central zone and an increase in time spent in the periphery (F(5,54) = 9.735, *p* < 0.0001) (Figure 3C,D), accompanied by a prolonged latency to the first entry into the center (F(5,54) = 2.456, *p* = 0.0446) (Figure 3B). In parallel, a trend toward increased immobility duration was observed (F(5,54) = 2.992, *p* = 0.0187) (Figure 3E), confirming the anxiogenic effect and avoidance behavior induced by SCO.

Chronic CEE treatment counteracted these effects in a dose-dependent manner. The 3 mg/L dose most effectively restored exploratory behaviors by increasing the time spent in the center (Figure 3D) and reducing latency compared with SCO (* *p* = 0.031) (Figure 3B), while also diminishing immobility (Figure 3E). Administration of 1 mg/L produced moderate improvements, whereas 10 mg/L yielded intermediate effects, placing values between controls and the SCO group. GAL exerted a robust anxiolytic effect, comparable to that of CEE at 3 mg/L, thereby confirming the pharmacological validity of the model.

In contrast, basic locomotor parameters, such as total distance traveled (F(5,54) = 2.256, *p* = 0.0618) (Figure 3F), did not show significant differences between groups, suggesting that the observed changes specifically reflect anxiety and center-avoidance behavior, without alterations, in general, motor activity.

### 2.2. Effects of CEE on Cognitive Performance

#### 2.2.1. Y Maze

**Figure 4:**
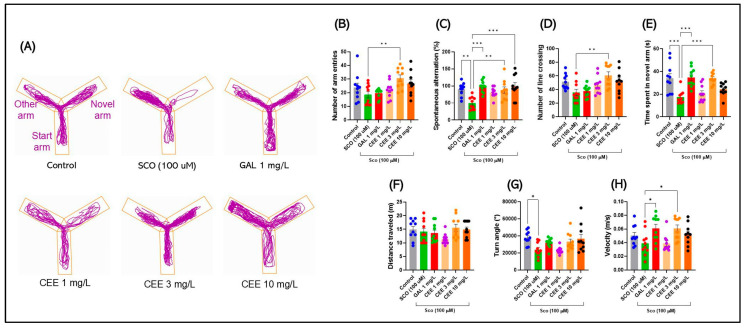
Effects of scopolamine (SCO) and *Calendula officinalis* ethanolic extract (CEE) in the Y-maze test. (**A**) Representative track plots for each group: Control, SCO (100 μM), Galantamine (GAL, 1 mg/L), and CEE 1–10 mg/L. (**B**–**H**) Quantified parameters: (**B**) Number of arm entries; (**C**) Spontaneous alternations (%); (**D**) Number of line crossings; (**E**) Time spent in the novel arm; (**F**) Total distance traveled; (**G**) Turn angle; (**H**) Mean swimming speed (Velocity). Data are expressed as mean ± SEM (n = 10 per group). * *p* < 0.05, ** *p* < 0.01, and *** *p* < 0.001.

Representative track plots in Figure 4A illustrate how SCO impaired spatial exploration in zebrafish. Compared with controls, SCO-exposed fish showed a clear tendency to avoid the novel arm, with exploratory patterns restricted to the familiar arms. In contrast, treatment with CEE, particularly at 3 mg/L, restored entries and time spent in the novel arm, indicating improved spatial memory. GAL produced a comparable effect, further confirming the pharmacological validity of the test. The 1 and 10 mg/L doses produced intermediate effects, with a more balanced exploration of all three arms compared with the SCO-only group.

Acute SCO administration induced clear deficits in the Y-maze, confirming the validity of the cholinergic model. A significant overall treatment effect was found (F(5,54) = 3.706, *p* = 0.0059). Post hoc testing showed that the CEE 3 mg/L group exhibited significantly more arm entries than the SCO group (*p* = 0.0084), whereas the SCO group did not differ significantly from the Control. However, post hoc analysis revealed significant decreases compared with the CEE 3 mg/L group (*p* = 0.0084), suggesting a selective reduction in exploratory behavior.

Moreover, SCO produced a marked deficit in spatial working memory, reflected by a reduction in the percentage of spontaneous alternations (F(5,53) = 6.019, *p* = 0.0034 vs. Control) (Figure 4C). SCO also substantially decreased the time spent in the novel arm (F(5,54) = 9.373, *p* < 0.0001; *** *p* = 0.0002 vs. Control) (Figure 4E), confirming impaired spatial memory. General locomotor parameters were less affected: total distance traveled did not differ significantly between groups (F(5,54) = 1.883, *p* = 0.1126) (Figure 4F), and swimming speed was unaltered relative to controls (Figure 4H), although intergroup differences were recorded (F(5,54) = 3.698, *p* = 0.0060). SCO also reduced turning angle (F(5,54) = 4.350, *p* = 0.0021; *p* = 0.0197 vs. Control) (Figure 4G), suggesting diminished cognitive flexibility, and decreased the number of line crossings (F(5,54) = 4.895, *p* = 0.0009), although not significantly compared with controls (Figure 4D).

Chronic CEE treatment exerted protective and procognitive effects in a dose-dependent manner. The 3 mg/L dose induced the most robust improvements, increasing the number of arm entries above control levels and restoring the percentage of spontaneous alternations to values comparable with controls (** *p* = 0.0078 vs. SCO; ns vs. Control) (Figure 4B,C). Furthermore, CEE 3 mg/L normalized preference for the novel arm (** *p* = 0.0003 vs. SCO) (Figure 4E), with an effect comparable to GAL.

The 1 mg/L dose produced moderate effects: it increased the time spent in the novel arm (** *p* = 0.0054 vs. Control) (Figure 4E) and partially improved memory, though without significant differences versus SCO. The 10 mg/L dose induced an intermediate recovery of turning angle, but this was not significant, and only produced a trend toward increased time spent in the novel arm (Figure 4G,E).

Regarding exploratory behavior, CEE 3 mg/L significantly increased the number of line crossings compared with SCO (*p* = 0.0016) (Figure 4D), indicating restoration of exploratory activity. Swimming speed showed dose-dependent differences between 1 and 3 mg/L (*p* = 0.0412), but no major locomotor alterations were detected (Figure 4H).

Overall, *C. officinalis* extract, particularly at 3 mg/L, effectively counteracted scopolamine-induced cognitive and exploratory deficits, confirming its procognitive profile and effects comparable to those of GAL.

#### 2.2.2. Novel Object Recognition (NOR)

**Figure 5:**
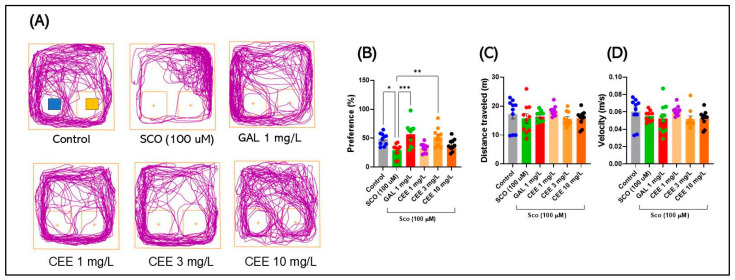
Effects of scopolamine (SCO) and *Calendula officinalis* ethanolic extract (CEE) in the Novel Object Recognition (NOR) test. (**A**) Representative track plots for each group: Control, SCO (100 μM), Galantamine (GAL, 1 mg/L), and CEE 1–10 mg/L. (**B**–**D**) Quantified parameters: (**B**) Preference for the novel object (%); (**C**) Total distance traveled (m); and (**D**) Mean swimming speed (m/s) (Velocity). Data are expressed as mean ± SEM (n = 10 per group). * *p* < 0.05, ** *p* < 0.01, and *** *p* < 0.001.

Representative track plots in Figure 5A highlight clear group differences in novel object preference. Control fish explored both stimuli in a balanced manner but showed a stronger tendency toward the novel object, reflecting intact recognition memory. In contrast, scopolamine-exposed zebrafish exhibited a marked reduction in interaction with the novel object and trajectories predominantly oriented toward the periphery, indicating a recognition deficit. Treatment with CEE, particularly at 3 mg/L, restored preference for the novel object, with repeated and prolonged trajectories in its vicinity, a profile comparable to that observed in the galantamine-treated group. The 1 and 10 mg/L doses produced intermediate effects, partially improving recognition behavior.

Acute SCO administration induced a robust recognition deficit in zebrafish, evidenced by a reduction in preference for the novel object (F(5,54) = 6.802, *p* < 0.0001) (Figure 5B). Compared with controls, the SCO group exhibited a significant decrease in this parameter (* *p* = 0.0299) (Figure 5B), confirming impairment of recognition memory. At the same time, locomotor parameters (distance traveled and mean swimming speed) did not differ significantly between groups (*p* > 0.05) (Figure 5C,D), indicating that the observed alterations were specifically related to cognitive processes rather than reduced motor activity.

Chronic CEE treatment counteracted these effects in a dose-dependent manner. Administration of 1 mg/L CEE increased preference for the novel object, although without reaching significance versus controls, but still showing improvement compared with SCO (Figure 5B). The 3 mg/L dose exerted the most pronounced procognitive effect, restoring performance to levels comparable to controls and similar to GAL (*p* = ns vs. Control; *** *p* = 0.0051 vs. SCO) (Figure 5B). At 10 mg/L, the extract produced only partial recovery, with a nonsignificant trend toward improvement (Figure 5B). GAL confirmed its role as a positive control, significantly increasing preference for the novel object compared with SCO (*** *p* = 0.0004) (Figure 5B).

Overall, these findings demonstrate that CEE, particularly at the 3 mg/L dose, has the capacity to restore recognition memory impaired by SCO, confirming a robust procognitive profile.

### 2.3. Effects of CEE on Biochemical Parameters in Zebrafish

#### 2.3.1. Acetylcholinesterase (AChE) Activity

**Figure 6:**
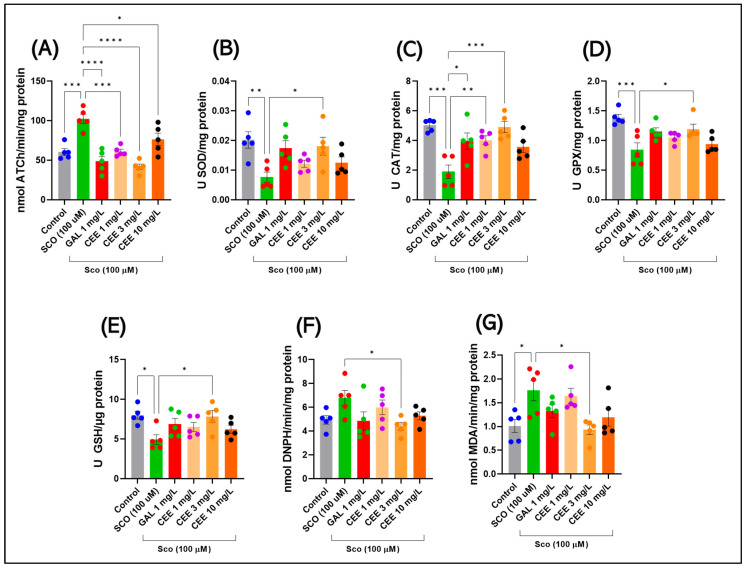
Effects of *Calendula officinalis* ethanolic extract (CEE) on biochemical markers in zebrafish exposed to scopolamine (SCO). (**A**) Acetylcholinesterase activity (AChE, nmol ATCh/min/mg protein); (**B**) Superoxide dismutase levels (SOD, U/mg protein); (**C**) Catalase (CAT, U/mg protein); (**D**) Glutathione peroxidase (GPx, U/mg protein); (**E**) Reduced glutathione (GSH, nmol/mg protein); (**F**) Protein carbonyls (nmol/mg protein); (**G**) Malondialdehyde (MDA, nmol/mg protein). Data are expressed as mean ± SEM (n = 5 per group). * *p* < 0.05, ** *p* < 0.01, *** *p* < 0.001, **** *p* < 0.0001.

Acute SCO administration induced a significant increase in brain acetylcholinesterase (AChE) activity (F(5,24) = 16.37, *p* < 0.0001) (Figure 6A). Compared with controls, the SCO group exhibited markedly elevated enzymatic activity (*** *p* = 0.0002), confirming the induction of a cholinergic deficit through accelerated degradation of acetylcholine (ACh).

Chronic CEE treatment reduced AChE activity in a dose-dependent manner. The 3 mg/L dose exerted the most pronounced inhibitory effect, significantly decreasing activity compared with SCO (**** *p* < 0.0001) (Figure 6A) to levels close to control. Administration of 1 mg/L produced a moderate reduction, while 10 mg/L induced an intermediate effect, maintaining values significantly lower than SCO (* *p* = 0.0251) but higher than CEE 3 mg/L (** *p* = 0.0016) (Figure 6A). GAL, used as a positive control, markedly reduced AChE activity relative to SCO (**** *p* < 0.0001), reaching values comparable to those of the control group. Interestingly, in direct comparison with CEE 10 mg/L, enzymatic activity was even lower (* *p* = 0.0139), confirming the robustness of its inhibitory effect.

Overall, these results indicate that CEE effectively modulates the cholinergic system, with an optimal profile at 3 mg/L, suggesting a mechanism of restoration of acetylcholine neurotransmission impaired by SCO.

#### 2.3.2. Antioxidant Enzymes (SOD, CAT, GPx)

Acute SCO administration induced a major imbalance in enzymatic antioxidant defenses. Superoxide dismutase (SOD) activity was significantly reduced compared with controls (F(5,24) = 4.118, *p* = 0.0077; *p* = 0.0088 vs. Control) (Figure 6B), confirming the induction of oxidative stress. Similarly, SCO strongly suppressed catalase (CAT) activity (F(5,24) = 8.419, *p* = 0.0001; * *p* = 0.0001 vs. Control) (Figure 6C) and glutathione peroxidase (GPx) activity (F(5,24) = 6.563, *p* = 0.0006; * *p* = 0.0004 vs. Control) (Figure 6D). These concomitant decreases highlight the impairment of enzyme-based antioxidant defenses, favoring the accumulation of reactive oxygen species and lipid peroxidation products.

By contrast, chronic CEE treatment exerted significant and dose-dependent protective effects. For SOD, only the 3 mg/L dose significantly improved activity compared with SCO (*p* = 0.0387) (Figure 6B), whereas 1 and 10 mg/L induced moderate but nonsignificant increases. In the case of CAT (Figure 6C), the 1 and 3 mg/L doses almost completely restored activity (** *p* = 0.0091 and * *p* = 0.0002 vs. SCO), while 10 mg/L produced a recovery trend (*p* = 0.0566). For GPx (Figure 6D), all CEE doses showed beneficial effects, with pronounced impact at 1 mg/L (*p* = 0.0441 vs. Control) and 3 mg/L (*p* = 0.0335 vs. SCO), and the 10 mg/L extract demonstrated the strongest protective effect (*p* = 0.0040 vs. Control).

As a pharmacological reference, galantamine partially normalized enzymatic activity. Its effect was significant, particularly for CAT (*p* = 0.0117 vs. SCO), but no notable differences compared with scopolamine were observed in the case of SOD and GPx.

#### 2.3.3. Glutathione (GSH) Levels

Acute SCO administration produced a significant reduction in brain GSH levels compared with controls (F(5,24) = 3.340, *p* = 0.0198; *p* = 0.0197 vs. Control) (Figure 6E). This decrease highlights the diminished endogenous antioxidant capacity and the accumulation of redox imbalances associated with the induction of oxidative stress.

Chronic CEE treatment exerted a partial protective effect on GSH levels (Figure 6E). The 3 mg/L dose produced a significant increase compared with SCO (*p* = 0.0299 vs. SCO), restoring values toward control levels. The 1 and 10 mg/L doses showed trends toward improvement but without statistical significance. GAL partially restored GSH levels, although the difference versus control was not significant (ns) (Figure 6E).

These results suggest that the antioxidant effects of CEE also involve protection of the glutathione system, with an optimal profile at the 3 mg/L dose.

#### 2.3.4. Oxidative Stress Markers (MDA, Protein Carbonyls)

Acute SCO administration induced a clear pro-oxidative profile. An increase in protein carbonyl levels was observed compared with controls (F(5,24) = 2.598, *p* = 0.0515) (Figure 6F), although the difference did not reach statistical significance. This trend suggests the onset of oxidative stress processes with impairment of neuronal protein structure and function. In parallel, SCO significantly increased MDA levels, a marker of lipid peroxidation (F(5,24) = 4.440, *p* = 0.0053; *p* = 0.0304 vs. Control) (Figure 6G), confirming compromised membrane integrity through free radical accumulation.

Chronic CEE treatment partially counteracted these alterations. At the protein level, the 3 mg/L dose significantly reduced SCO-induced carbonylation (*p* = 0.0481 vs. SCO) (Figure 6F), whereas the 1 and 10 mg/L doses induced only moderate, nonsignificant variations (Figure 6F). Regarding lipid peroxidation, administration of 3 mg/L CEE restored MDA values to levels close to controls (*p* = 0.0133 vs. SCO) (Figure 6G), while the 1 and 10 mg/L doses produced partial, nonsignificant effects. GAL exerted a comparable protective effect, reducing both protein carbonyls (Figure 6F) and MDA levels (Figure 6G), although differences versus SCO were not consistently significant.

These results indicate that CEE, particularly at the 3 mg/L dose, exerts a relevant antioxidant effect on both proteins and lipids, contributing to the maintenance of neuronal structural and functional integrity under SCO-induced oxidative stress.

### 2.4. Correlations Between Behavioral Parameters and Biomarkers

**Figure 7:**
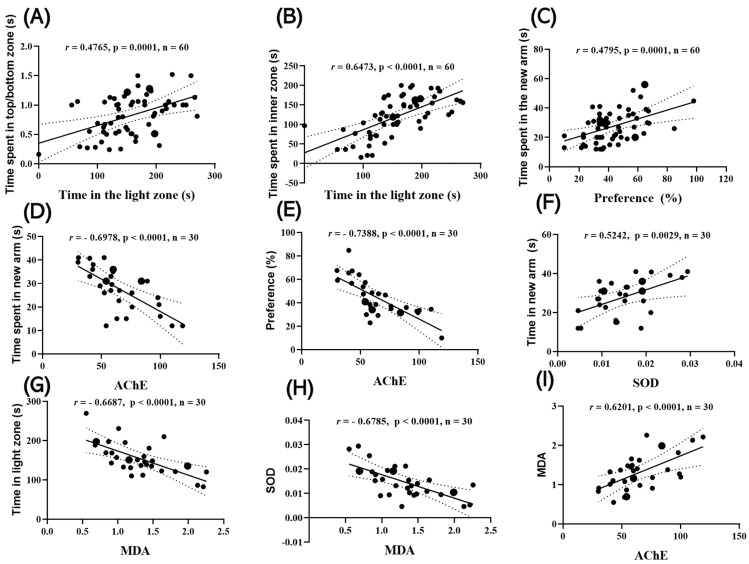
Correlations between behavioral and biochemical markers (Pearson). (**A**) NTT: Time in the upper zone vs. LDT: Time in light (r = 0.4765; *p* = 0.0001). (**B**) NAT: Time in the inner zone vs. LDT: Time in light (r = 0.6473; *p* < 0.0001). (**C**) Y-maze: Time in the novel arm vs. NOR: Preference (%) (r = 0.4795; *p* = 0.0001). (**D**) Y-maze: Time in the novel arm vs. AChE (nmol ATCh/min/mg protein) (r = −0.6978; *p* < 0.0001). (**E**) NOR: Preference (%) vs. AChE (r = −0.7388; *p* < 0.0001). (**F**) Y-maze: Time in the novel arm vs. SOD (U/mg protein) (r = 0.5242; *p* = 0.0029). (**G**) LDT: Time in light vs. MDA (nmol/mg protein) (r = −0.6687; *p* < 0.0001). (**H**) SOD vs. MDA (r = −0.6785; *p* < 0.0001). (**I**) MDA vs. AChE (r = 0.6201; *p* = 0.0003).Sample sizes: behavioral n = 10/group (6 groups; 60 XY pairs); behavior–biochemistry n = 5/group (paired subset; 30 XY pairs); biochemistry n = 5/group (30 XY pairs). Values represent Pearson’s r (two-tailed). Scatter plots include regression line and 95% CI.

Time spent in the light zone (LDT) was positively correlated with time spent in the upper zone in the NTT (r = 0.4765; 95% CI: 0.2533–0.6516; *p* = 0.0001) (Figure 7A) and with time spent in the inner zone in the NAT (r = 0.6473; 95% CI: 0.4708–0.7740; *p* < 0.0001) (Figure 7B). In addition, memory performance measures converged: NOR preference was positively correlated with time spent in the novel arm in the Y-maze (r = 0.4795; 95% CI: 0.2568–0.6538; *p* = 0.0001) (Figure 7C).

Time spent in the novel arm (Y-maze) was positively correlated with AChE activity (r = −0.6978, 95% CI; *p* < 0.0001) (Figure 7D). NOR preference was negatively correlated with AChE activity (r = −0.7388; 95% CI: −0.8455 to −0.5159; *p* < 0.0001) (Figure 7E), while time spent in the novel arm (Y-maze) was positively correlated with SOD activity (r = 0.5242; 95% CI: 0.2021–0.7439; *p* = 0.0029) (Figure 7F). MDA levels were negatively correlated with time spent in the light zone in the LDT (r = −0.6687; 95% CI: −0.8292 to −0.4063; *p* < 0.0001) (Figure 7G), and biochemically, MDA were inversely correlated with SOD (r = −0.6785; 95% CI: −0.8347 to −0.4212; *p* < 0.0001) (Figure 7H). and positively correlated with AChE (r = 0.6201; 95% CI: 0.3345–0.8013; *p* = 0.0003) (Figure 7I).

Taken together, lower anxiety (greater light exposure in LDT) and enhanced spatial exploration (Y-maze novel arm entry) were associated with a stronger antioxidant profile (SOD ↑) and reduced oxidative/cholinergic stress (MDA ↓, AChE ↓). Conversely, increased AChE activity and elevated MDA were linked to poorer memory performance and heightened anxiety. All correlations remained significant after correction for multiple comparisons (FDR, *p* < 0.05).

## 3. Discussion

Our study aimed to investigate the neuroprotective potential of chronic administration of *Calendula officinalis* ethanolic extract (CEE; 1, 3, and 10 mg/L) against scopolamine (SCO, 100 µM)-induced anxiety-like behaviors and cognitive deficits in zebrafish. The choice of this experimental model was based on the fact that SCO is a well-established cholinergic agent used to induce memory impairment and anxiety in both rodents and fish, thereby partially reproducing pathogenic mechanisms implicated in human neurocognitive disorders [4,17]. On the other hand, *C. officinalis* is recognized for its rich phytochemical profile, including flavonoids, triterpenoids, and carotenoids—compounds with well-documented antioxidant and anti-inflammatory properties [12,14,18]. However, experimental evidence regarding its effects on cognitive functions and anxiety-like behavior in animal models remains scarce.

Ethanolic extraction (70%) was selected in the present study as it provides an optimal balance between polarity and biocompatibility, allowing the simultaneous recovery of both hydrophilic phenolic acids and flavonoids, as well as lipophilic carotenoids and triterpenoids. Previous comparative analyses have demonstrated that ethanolic extracts of *C. officinalis* yield higher total flavonoid content and superior antioxidant activity (e.g., FRAP, DPPH, ABTS) compared to aqueous infusions or extracts obtained with pure organic solvents [18,19]. Ethanol is also regarded as a “green” solvent, with proven safety in pharmaceutical and nutraceutical applications, in contrast to solvents such as methanol or chloroform, which, although effective for extracting certain phytochemicals, are toxic and translationally irrelevant [20,21,22]. Therefore, the use of ethanolic extracts enhances both the reproducibility of phytochemical profiles and the translational value of the findings, supporting their investigation in behavioral and neurochemical models of cognitive and anxiety-related disorders. In this context, the current study sought to provide an integrated behavioral and biochemical analysis of the role of CEE in counteracting SCO-induced effects.

Galantamine (GAL), an alkaloid used clinically for the symptomatic treatment of Alzheimer’s disease (AD), acts through reversible inhibition of AChE and modulation of nicotinic receptors, thereby facilitating cholinergic neurotransmission [23]. In our study, it was included as a positive control to validate the SCO-induced model. Its anxiolytic and procognitive effects were comparable to those observed with CEE at 3 mg/L, confirming both the pharmacological validity of the model and the translational relevance of CEE.

Over the past two decades, zebrafish have emerged as a robust translational model for investigating cognitive functions and neuropsychiatric disorders. The cholinergic system in zebrafish exhibits an organization comparable to that of mammals, including muscarinic and nicotinic receptors that regulate attention and memory [24,25]. Exploratory and anxiety-related behaviors (e.g., NTT, Y-maze) are well characterized and display sensitivity to pharmacological agents also used in rodent models, thereby providing both predictive and construct validity [26,27]. Moreover, zebrafish respond to SCO with memory impairments and anxiety-like behaviors similar to those observed in mice and rats. These effects are reversed by established procognitive agents such as GAL or donepezil, further confirming the convergence of cholinergic mechanisms [26,28]. This parallelism with mammalian models underscores the utility of zebrafish for preclinical screening of neuroactive compounds, while also reducing experimental costs and time.

Our results demonstrated that chronic administration of CEE significantly attenuated anxiety-like behaviors and cognitive deficits induced by SCO in zebrafish. These beneficial effects were dose-dependent, with the optimal profile observed at 3 mg/L, which nearly restored both behavioral and biochemical parameters to control levels. At the neurochemical level, CEE normalized AChE activity and reestablished antioxidant balance by increasing SOD, CAT, GPx, and GSH levels, while simultaneously reducing oxidative stress markers such as MDA and protein carbonyls. Correlation analysis confirmed a direct association between the improvement of cognitive and anxiolytic performance and the amelioration of cholinergic and redox status, supporting the hypothesis of an integrated mechanism of action.

An interesting observation in our study is that the intermediate dose of CEE (3 mg/L) consistently produced the most pronounced behavioral and biochemical effects, whereas the higher dose (10 mg/L) often showed only moderate efficacy. Such bell-shaped or inverted U-shaped dose–response patterns are frequently reported for botanical extracts and polyphenolic compounds within the framework of hormesis [29,30,31,32]. Several mechanisms may account for this phenomenon, including the coexistence of multiple phytochemicals with potentially antagonistic actions at higher concentrations, the emergence of pro-oxidant activity at elevated doses, or nonspecific binding and receptor desensitization. Notably, *C. officinalis* contains flavonoids such as quercetin and rutin [33], both of which have documented hormetic (biphasic) dose-dependent responses. Although further studies are needed, these results support the hypothesis that the optimal efficacy of complex botanical extracts occurs within a specific “dose window,” rather than increasing linearly with concentration.

These findings are consistent with recent studies reporting that phytochemical extracts ameliorate SCO-induced cognitive and behavioral deficits in experimental models. For example, a study published in *Fish Physiology and Biochemistry* showed that betanin treatment protected zebrafish against SCO (100 µM)-induced memory impairments and oxidative stress by normalizing AChE activity, enhancing BDNF expression, and reducing MDA levels [34].

Similarly, dietary supplementation with lutein as an antioxidant significantly reduced spatial learning and memory impairments in both zebrafish and mice, primarily through AChE inhibition and reduced lipid peroxidation [35]. In rodent models, several studies have further confirmed the relevance of cholinergic and antioxidant mechanisms in protecting against SCO-induced cognitive deficits. For instance, administration of febuxostat, either alone or in combination with donepezil, improved cognitive performance and reduced oxidative stress markers. These effects suggest a potential translational value of pharmacological combinations that simultaneously modulate purine metabolism and cholinergic neurotransmission by targeting the thioredoxin-interacting protein (TXNIP)/NOD-like receptor (NLRP3) inflammasome pathway [36]. Moreover, astaxanthin, a carotenoid with potent antioxidant properties, demonstrated neuroprotective effects in a murine SCO-induced AD model. Its administration enhanced catalase activity and GSH levels while reducing hippocampal NF-κB and MMP-9 expression, highlighting the complex interplay between oxidative stress, inflammation, and cognitive impairment [37]. Along the same lines, gentisic acid—a phenolic compound widely distributed in plants—exerted notable procognitive effects in mice by protecting against cholinergic dysfunction and preserving neuronal integrity. These benefits were associated with reduced lipid peroxidation and improved antioxidant status [38].

Evidence from the literature indicates that *C. officinalis* possesses relevant neuropsychopharmacological potential, although studies directly addressing its effects on cognitive functions and anxiety-related behaviors remain limited. Hydroalcoholic extracts of *Calendula* have been reported to exert significant AChE inhibitory activity and robust antioxidant properties, both key mechanisms for memory protection and anxiety reduction [14]. In addition, there is evidence that certain fractions of *Calendula* may interact with target enzymes involved in cholinergic neurotransmission, suggesting an indirect role in the amelioration of cognitive and anxiety-related disturbances [16].

Our findings are consistent with emerging evidence regarding the neuroprotective potential of *C. officinalis* in neurodegenerative disorders. In an MPTP-induced Parkinson’s disease (PD) model in zebrafish, ethanolic *Calendula* extract demonstrated remarkable effects on the protection of dopaminergic neurons and neural vasculature, concomitant with the restoration of neurodevelopmental gene expression and improvement of motor deficits. The anti-PD effect of ethanolic *Calendula* extract was associated with autophagy activation, mediated by the upregulation of Pink, Ulk2, Atg7, and Lc3b, which facilitated the degradation of α-synuclein aggregates and dysfunctional mitochondria. Molecular docking analyses revealed stable interactions between the flavonoids identified in the extract and key autophagy regulators, reinforcing the hypothesis that *C. officinalis* exerts neuroprotective effects through an integrated flavonoid–autophagy–dopaminergic mechanism [39]. Furthermore, in PD zebrafish models, *Calendula* extracts nearly restored the morphology and density of dopaminergic neurons, an effect attributed to bioactive compounds such as 3,4-dicaffeoylquinic acid, isorhamnetin 3-O-glucoside, and calenduloside E. These molecules appear to act by activating the PI3K/Akt pathway and inhibiting ERK/Hsp90α signaling, mechanisms that support both neuronal protection and the improvement of cognitive and anxiolytic behaviors [40,41]. Moreover, methanolic extracts of *Calendula* flowers have shown protective effects in rat models of neurotoxicity induced by monosodium glutamate and 3-nitropropionic acid, by reducing oxidative stress and preserving hippocampal and striatal neurons. In experimental models of AlCl_3_-induced AD, doses of 100–300 mg/kg of *Calendula* extract exerted dose-dependent neuroprotective effects, with maximal efficacy at the highest dose [42].

Beyond the neurobehavioral findings, *C. officinalis* also modulates key nodes of redox–inflammatory homeostasis. In an in vivo wound-healing model, a standardized lipophilic extract (dominated by 1-heptatriacotanol and the sesquiterpene isochiapin B) accelerated epithelialization and collagen synthesis, accompanied by increased AMPK and HIF-1α activity and decreased MMP-9 expression, against a background of high antioxidant capacity. Molecular docking analyses revealed a strong affinity of isochiapin B for MMP-9 (along with relevant interactions with AMPK, HIF-1α, TNF-α, and VEGF), suggesting its potential to attenuate inflammatory cascades [43]. Considering the convergence of these pathways with mechanisms implicated in SCO-induced cognitive and anxiety-like deficits (oxidative stress, MMP activity, pro-inflammatory signaling), these findings support the hypothesis that the lipophilic constituents of Calendula contribute to the procognitive and anxiolytic effects observed in our model through an integrated cholinergic–redox–inflammatory mechanism. Nevertheless, it should be noted that these data originate from a cutaneous model; extrapolation to the CNS remains inferential, although the overlap of pathways (AMPK/HIF-1α/MMP-9/TNF-α) provides biological plausibility for the observed neuroprotective effects. The neuroprotective relevance of *C. officinalis* is further supported by recent preclinical evidence. Gold nanoparticles synthesized via a green approach using *Calendula* extract demonstrated high antioxidant capacity and favorable cellular tolerability. When administered intravenously at doses of 10–250 µg/kg, these nanoparticles reduced cerebral infarct volume, serum LDH activity, and MDA levels, while preserving the BBB and improving memory and learning functions in a rat ischemia–reperfusion model [44].

Taken together, the available evidence indicates that bioactive metabolites of *C. officinalis* may contribute to neurovascular protection, conferring the plant with translational potential in the management of cognitive and anxiety-related disorders. Corroborated with our findings on the amelioration of SCO-induced anxiety and memory deficits, these results suggest an extended translational spectrum that applies both to cholinergic-type cognitive disturbances and to pathologies associated with α-synuclein aggregation. Overall, the literature supports the hypothesis that the anxiolytic and procognitive effects observed in zebrafish in our study are driven by a combination of cholinergic modulation and the antioxidant and anti-inflammatory properties of CEE.

Our study, however, presents certain limitations, including the exclusive use of the zebrafish model, reliance on a crude ethanolic extract, and biochemical analysis restricted to redox and cholinergic markers. In addition, the short duration of treatment and the lack of sex-based analyses limit the generalizability of the results. Although we used a balanced sex ratio (1:1 males/females), the study was not designed to analyze sex-specific differences. The reduced number of individuals per sex (n ≈ 5) does not allow for a robust analysis, and conclusions would be potentially misleading. Future studies with larger sample sizes and a factorial design (treatment × sex) are needed to determine whether C. officinalis extract exerts differential effects in males and females. Another methodological limitation relates to the extraction protocol. Although only a relatively small amount of plant material (1.5 g of fresh petals per extraction) was used, the applied turbo-extraction yielded a sufficiently concentrated extract for the behavioral assays. Importantly, the residual ethanol concentration in aquarium water (<0.015% *v*/*v*) was far below the threshold reported to affect zebrafish behavior [45], confirming that the observed effects can be attributed to the extract itself rather than to the vehicle. Furthermore, another limitation concerns the discrepancy between the sample size used for behavioral evaluations and that used for biochemical analyses. All fish (n = 10/group) were subjected to behavioral testing, whereas only a random subset of five brains per group was processed for biochemical determinations. This choice was made in accordance with the ethical 3R principles (Replacement, Reduction, Refinement), aiming to minimize the number of animals sacrificed while maintaining statistical robustness. Similar approaches, with n = 5 for biochemical analyses, have been reported in zebrafish studies evaluating oxidative stress and neurochemical markers [46,47]. Although this represents a methodological limitation, the consistency of behavioral and biochemical findings supports the validity of our conclusions. Future research should aim to validate these findings in mammalian models, isolate the active fractions, and further explore the molecular pathways involved in neuroprotection. Extending the duration of administration and testing innovative formulations could strengthen the translational potential of *C. officinalis* in cognitive and anxiety-related disorders.

Overall, our findings demonstrate that chronic administration of CEE attenuates scopolamine-induced anxiety and cognitive deficits in zebrafish through mechanisms involving both the restoration of cholinergic function and the reduction in oxidative stress. These observations are consistent with recent literature confirming the neuroprotective potential of *Calendula* in models of neurotoxicity, AD, PD, and cerebral ischemia, with its effects largely attributed to flavonoids and triterpenoids exhibiting antioxidant, anti-inflammatory, and autophagy-modulating activities. Although further studies are required to validate these findings in mammalian models and to identify the specific active constituents responsible, the present results support the hypothesis that *C. officinalis* represents a promising phytotherapeutic resource with translational relevance for cognitive and anxiety-related disorders.

## 4. Materials and Methods

### 4.1. Plant Material

The petals of *Calendula officinalis* (marigold) flowers were collected from the Sibiu region, Romania. Samples were stored at −70 °C until use, and moisture content was determined using a moisture analyzer (MAC 210/NP, Radwag, Radom, Poland).

All chemical reagents used were of analytical grade.

### 4.2. Preparation of Extracts

The marigold flower petals (*Calendula officinalis*) were collected from the Sibiu region, Romania, and stored at −70 °C until analysis. Extraction was performed in 70% ethanol at a solvent/sample ratio of 20:1, using turbo-extraction with a high-shear homogenizer (T 18 digital Ultra-Turrax^®^, IKA, Staufen im Breisgau, Germany) at 6000 rpm, room temperature, for 10 min. Subsequently, the extracts were centrifuged at 8000 rpm, 4 °C, for 10 min (Universal 320-R refrigerated centrifuge, Hettich, Tuttlingen, Germany), and the supernatant was collected for further use in biological assays. The extract used in this study was previously characterized in another study of our research group, with the following reported values of bioactive compounds [48]: total polyphenol content—1088.31 ± 3.34 mg GAE/100 g DW; total flavonoids—2253.73 ± 7.26 mg QE/100 g DW; condensed tannins—426.62 ± 4.19 mg CE/100 g DW; FRAP antioxidant activity—479.46 ± 2.77 mg AAE/100 g DW; and DPPH radical scavenging activity—7.62 ± 0.04%.

### 4.3. Animals

In this study, we used adult wild-type short-fin zebrafish (*Danio rerio*), aged 4–6 months, with a body length of 3–4 cm and a weight of approximately 0.4–0.6 g, in a balanced sex ratio (1:1). Animals were obtained from a licensed supplier and acclimatized for 14 days prior to the initiation of the experimental protocol.

During acclimatization, fish were maintained in 70 L tanks, 10 individuals per aquarium, in dechlorinated water treated with Tetra AquaSafe (Tetra, Melle, Germany), renewed daily. Environmental parameters were kept constant: temperature 27 ± 1 °C, pH 7.0–7.5, dissolved oxygen 8 ± 1 mg/L, conductivity 1500–1600 µS/cm, and ammonium and nitrite concentrations <0.001 mg/L. A 14 h light/10 h dark photoperiod was applied to simulate the natural circadian rhythm. Feeding was performed twice daily (07:00 and 16:00) with commercial flakes (Norwin, Gadstrup, Denmark), in amounts adjusted to be fully consumed within 10 min.

All procedures were conducted in accordance with Directive 2010/63/EU and were approved by the Ethics Committee of Alexandru Ioan Cuza University of Iasi (approval no. 1714/06.07.2023).

### 4.4. Treatment Administration

**Figure 8:**
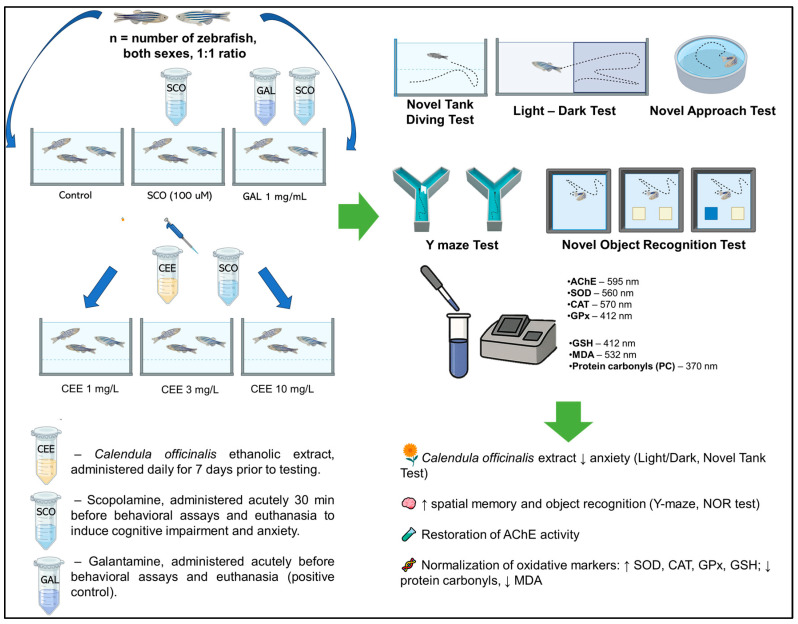
Experimental design. Adult zebrafish were randomly assigned to seven groups: Control, SCO (100 µM), CEE 1–10 mg/L + SCO, GAL (1 mg/L), and SCO + GAL. CEE was administered daily for 7 days, followed by acute SCO exposure (100 µM, 30 min). GAL was applied acutely (3 min) as positive control. Behavioral tests (NTT, LDT, NAT, Y-maze, NOR) were conducted, and brain samples were analyzed for cholinergic (AChE) and oxidative stress markers (SOD, CAT, GPx, GSH, protein carbonyls, MDA).

After the acclimatization period, fish were randomly allocated into seven experimental groups (Figure 8) (n = 10/group): I. Control: fish maintained under standard conditions without treatment. II. SCO: scopolamine (100 µM, immersion for 30 min before testing). III. CEE 1 + SCO: *Calendula officinalis* ethanolic extract (CEE) 1 mg/L, administered daily throughout the experimental period (22 days), including a pretreatment phase of 7 days prior to SCO (100 µM). IV. CEE 3 + SCO: CEE 3 mg/L, administered daily for 22 days, with SCO applied after the first 7 days of exposure. V. CEE 10 + SCO: CEE 10 mg/L, administered daily for 22 days, with SCO applied after the first 7 days of exposure. VI. GAL: galantamine (1 mg/L, immersion for 3 min before testing). VII. SCO + GAL (positive reference): SCO (100 µM, 30 min) followed by GAL (1 mg/L, 3 min) immediately before testing. The concentrations of SCO and GAL used in this study were selected based on our previous reports [18,49]. In all experimental groups where CEE, GAL, or SCO + GAL were administered, the residual ethanol concentration in aquarium water was <0.015% *v*/*v*, corresponding to the maximum dose of 10 mg/L of CEE. This concentration is well below the threshold at which ethanol is known to affect zebrafish behavior [47] and therefore cannot account for the differences observed between groups.

The extract was dissolved in aquarium water at the indicated concentrations and renewed daily with the water change (08:00 h) for seven consecutive days. Fish in groups also exposed to SCO received acute treatment (100 µM, immersion for 30 min) prior to anxiety- and locomotion-related tests (NTT, LDT, NAT) and 30 min before the test phase in cognitive paradigms (Y-maze and NOR). For tasks involving both training and test phases (Y-maze, NOR), SCO was also administered 30 min after the training session to induce an amnestic deficit. GAL (1 mg/L) was administered by immersion for 3 min immediately before testing, either as a single treatment (GAL group) or in combination with SCO (SCO + GAL group). Group allocation was randomized, and experimenters involved in behavioral and statistical analyses were blinded to treatment conditions.

The same cohort of zebrafish from each group (n = 10) was sequentially subjected to all behavioral paradigms. Behavioral testing began on day 7 with the Novel Tank Diving Test (NTT), followed by a two-day interval before the NAT (day 10). After another two-day interval, the LDT was performed on day 13, followed by a further two-day interval before the Y-maze test on day 16. One day later (day 17), fish were rested, and then the NOR task was conducted over four consecutive days (days 18–21). On day 22, fish received the final treatment and were euthanized in a manner similar to our previous report [18].

### 4.5. Behavioral Analysis

Behavioral tests were conducted in a quiet room under controlled lighting conditions between 09:00 and 17:00. Each fish was tested individually, with an interval of at least two days between trials to minimize the influence of repeated stress. Sessions were video-recorded using an overhead digital camera (Logitech HD Webcam C922 Pro Stream, Logitech International S.A., Lausanne, Switzerland), and behavioral analysis was performed with ANY-maze^®^ software v7.44 (Stoelting Co., Wood Dale, IL, USA).

#### 4.5.1. Novel Tank Diving Test (NTT)

To assess anxiety-like behavior, fish were individually introduced into a transparent trapezoidal tank (~1.5 L capacity; dimensions 15 × 24 × 7 × 29 cm), virtually divided into upper and lower zones, as described by Cachat et al. [27]. Following exposure to SCO or treatment, each animal was recorded for 6 min. The parameters analyzed included: latency to the first ascent, total time spent in the upper/lower zones, number of entries into the upper zone, distance traveled, average swimming speed, and duration of freezing behavior.

#### 4.5.2. Light/Dark Test (LDT)

The Light/Dark Test (LDT) was used to assess anxiety-like behavior, based on the natural preference of zebrafish for dark environments [18,50]. The experimental arena (55 × 9.5 × 9.5 cm) was divided into two equal compartments, one illuminated and the other covered with opaque black foil. Water temperature was maintained at 27 ± 1 °C. Each fish was placed in the center and observed for 5 min. The parameters analyzed included: time spent in the light zone, time spent in the dark zone, light zone preference, distance traveled, and average swimming speed.

#### 4.5.3. Novel Approach Test (NAT)

To evaluate responses to novel stimuli and anxiety-like behavior, an opaque circular tank (34 cm diameter, 15 cm wall height, water depth 6 cm, 27 ± 1 °C) was used, in accordance with the protocol previously described by Hamilton [10]. A novel object (a multicolored LEGO figurine, 5 cm) was placed in the center of the arena. The central zone (10 cm diameter) was defined as the “zone of interest,” while the remainder of the arena was considered the peripheral zone. Each fish was recorded for 5 min. The parameters analyzed included: time spent in the central zone, time spent in the periphery, latency to first approach, total distance traveled, and duration of immobility.

#### 4.5.4. Y-Maze Test

To assess spatial memory and novelty response, a Y-shaped maze (arms measuring 25 × 8 × 15 cm, with 120° angles) was used, with the external walls covered by geometric visual cues, as previously described by Cognato et al. [26]. During the training phase (5 min), fish had access only to the start arm and the familiar arm, while the novel arm was blocked. After a 1 h retention interval and exposure to SCO, fish were reintroduced into the maze for the test phase (5 min), with access to all three arms. The parameters analyzed included: number of arm entries, percentage of spontaneous alternations, time spent in the novel arm, number of line crossings, total distance traveled, swimming velocity, and turning angles.

#### 4.5.5. Novel Object Recognition (NOR)

To evaluate recognition memory, a cubic aquarium (30 × 30 × 30 cm) with opaque external walls, a white bottom, and a water level of 5 cm was used, in accordance with a previous study [46]. The protocol established by Stefanello et al. [51] included three phases: (I) Habituation: three days of exposure, with two 5 min sessions per day and a 5 h interval between sessions; (II) Training (day 4): exposure to two identical objects (yellow cubes, 2.5 cm) for 10 min; and (III) Test (day 5): after a 1 h retention interval and SCO exposure, one of the familiar objects was replaced with a novel object (blue cube). Each fish was recorded for 10 min during the test session. The parameters analyzed included preference (%) = (T_novel − T_familiar)/(T_novel + T_familiar), total distance traveled, and swimming velocity.

### 4.6. Determination of Biochemical Parameters

Brain tissues for biochemical assays were collected from the same animals that had previously undergone the full behavioral tests, ensuring paired behavioral–biochemical data. From each group, a random subset of five brains was selected for biochemical analyses in accordance with the 3R ethical principles. After completion of behavioral testing, fish were individually transferred to glass containers with cold water (2–4 °C) for 10 min to induce anesthesia, followed by euthanasia through rapid decapitation, in accordance with approved protocols [52]. Brains were immediately extracted as previously described [53], individually weighed (≈3–6 mg), and stored in 0.5 mL microtubes at −20 °C until processing. On the following day, brain samples from each experimental group were homogenized at a 1:10 (w/v) ratio (mg tissue per µL buffer) in cold phosphate buffer (0.1 M potassium phosphate, pH 7.4, containing 1.15% KCl) using a bead homogenizer (Mikro-Dismembrator U, Sartorius, NY, USA). Homogenates were centrifuged at 14,000 rpm for 15 min at 4 °C (Eppendorf 5417R refrigerated centrifuge, Hamburg, Germany), and the supernatants were used for biochemical assays. All reagents were of analytical grade, and determinations were performed in duplicate for each sample.

Total protein concentration was measured using the Bradford method [54], with bovine serum albumin (BSA; Sigma-Aldrich, Darmstadt, Germany) as the standard and Coomassie Brilliant Blue G-250 (AppliChem, Darmstadt, Germany) as the dye. Absorbance was read at 595 nm with a Beckman Coulter DU 730 Life Science UV-VIS spectrophotometer (Brea, CA, USA).

AChE (EC 3.1.1.7) activity was determined by the Ellman method [55], using acetylthiocholine iodide (Sigma-Aldrich, Germany) and 5,5′-dithiobis(2-nitrobenzoic acid, DTNB; Sigma-Aldrich, Germany). Absorbance was read at 412 nm (Beckman Coulter DU 730). Results were expressed as nmol substrate hydrolyzed/min/mg protein.

SOD (EC 1.15.1.1) activity [56] was evaluated by the inhibition of nitro blue tetrazolium (NBT; AppliChem, Darmstadt, Germany) reduction by the superoxide radical generated in the presence of riboflavin (Sigma-Aldrich, Germany) under fluorescent light (Philips, Amsterdam, The Netherlands), in a medium containing disodium EDTA (Carl Roth, Karlsruhe, Germany). Absorbance was read at 560 nm (Beckman Coulter DU 730). CAT (EC 1.11.1.6) activity was determined by the modified Sinha method [57] using potassium dichromate (Merck, Darmstadt, Germany) and glacial acetic acid (Merck, Germany). Absorbance was measured at 570 nm (Beckman Coulter DU 730), and results were expressed as µmol H_2_O_2_ decomposed/min/mg protein. GPx (EC 1.11.1.9) activity [58] was determined by measuring the oxidation of reduced glutathione (GSH; Sigma-Aldrich, Germany) in the presence of hydrogen peroxide (Merck, Germany). Residual GSH was quantified by reaction with DTNB (Sigma-Aldrich, Germany). Absorbance was read at 412 nm (Beckman Coulter DU 730). Results were expressed as µg GSH oxidized/min/mg protein.

GSH levels were determined by the DTNB method [59] (Sigma-Aldrich, Germany), with absorbance read at 412 nm (Beckman Coulter DU 730), and expressed as µmol/mg protein. MDA, a marker of lipid peroxidation, was measured using the TBARS method [60], with thiobarbituric acid (TBA; Sigma-Aldrich, Germany) and perchloric acid (Merck, Germany). Samples were incubated at 95 °C for 60 min (Thermomixer F1.5, Eppendorf, Germany). The MDA–TBA adduct was measured at 532 nm (Beckman Coulter DU 730) and expressed as nmol/mg protein. Protein carbonyl content [61] was determined by reaction with 2,4-dinitrophenylhydrazine (DNPH; Sigma-Aldrich, Germany), followed by washes with ethanol/ethyl acetate (Merck, Germany). The pellet was solubilized in 6 M guanidine (Sigma-Aldrich, Germany). Absorbance was measured at 370 nm (Beckman Coulter DU 730) and expressed as nmol DNPH/mg protein.

All enzymatic assays (AChE, SOD, CAT, GPx) were conducted under conditions previously validated for zebrafish brain homogenates to ensure linearity with respect to protein concentration and reaction time [18,53].

### 4.7. Statistical Analysis

Data are presented as mean ± standard error of the mean (SEM). The effect of treatment on each parameter was evaluated using one-way ANOVA with six levels (Control, SCO 100 µM, GAL 1 mg/L, CEE 1, 3, and 10 mg/L). Homogeneity of variances was assessed with Brown–Forsythe and Bartlett’s tests, and the outcomes of these tests are reported alongside the ANOVA results. Post hoc comparisons between groups were performed using Tukey’s test. To assess the dose–response trend of CEE (1, 3, and 10 mg/L), a priori linear contrasts were applied.

Correlations between variables were analyzed using Pearson’s correlation coefficient (two-tailed), with r, 95% CI, and *p* reported. Behavioral–behavioral correlations were based on n = 10/group × 6 groups = 60 XY pairs; behavioral–biochemical correlations on n = 5/group × 6 groups = 30 XY pairs (paired subset); and biochemical–biochemical correlations on n = 5/group × 6 groups = 30 XY pairs. For datasets with multiple correlation tests, *p* values were adjusted using the FDR (Benjamini–Hochberg) method. Adjusted *p* significance levels are marked in figures (*** *p* < 0.001; ** *p* < 0.0001). Scatter plots include linear regression lines and 95% CI. The significance level was set at α = 0.05. Given the nearly balanced design (equal group sizes), ANOVA/Tukey is considered robust to moderate violations of variance homogeneity; nevertheless, Brown–Forsythe and Bartlett’s test results are reported for transparency. All analyses were performed using GraphPad Prism v9.4 (GraphPad Software, San Diego, CA, USA).

## 5. Conclusions

Our study demonstrates that the ethanolic extract of *Calendula officinalis* exerts anxiolytic and procognitive effects in the scopolamine zebrafish model by normalizing acetylcholinesterase activity and restoring oxidative balance. These findings, together with literature evidence on the neuroprotective roles of flavonoids and triterpenoids, suggest that *C. officinalis* holds relevant translational potential for cognitive and anxiety-related disorders. However, validation in mammalian models and the identification of bioactive fractions remain essential steps to confirm and extend these observations. Overall, our results suggest that CEE exerts its most pronounced effects at an intermediate dose (3 mg/L), consistent with an inverted U-shaped dose–response pattern frequently reported for botanical polyphenols. Future studies should further investigate this dose window to optimize the therapeutic potential of *C. officinalis* extract.

## Data Availability

The data are contained within this article.

## Abbreviations

The following abbreviations are used in this manuscript:

CEE	*Calendula officinalis* ethanolic extract
SCO	Scopolamine
GAL	Galantamine
AChE	Acetylcholinesterase
AD	Alzheimer’s disease
PD	Parkinson’s disease
MCI	Mild cognitive impairment
BBB	Blood–brain barrier
NTT	Novel Tank Diving Test
LDT	Light/Dark Test
NAT	Novel Approach Test
NOR	Novel Object Recognition
BDNF	Brain-derived neurotrophic factor
SOD	Superoxide dismutase
CAT	Catalase
GPx	Glutathione peroxidase
GSH	Reduced glutathione
MDA	Malondialdehyde
ROS	Reactive oxygen species
FRAP	Ferric reducing antioxidant power
DPPH	2,2-diphenyl-1-picrylhydrazyl radical scavenging assay
ABTS	2,2′-azino-bis(3-ethylbenzothiazoline-6-sulfonic acid) radical scavenging assay
TBARS	Thiobarbituric acid reactive substances
DNPH	2,4-dinitrophenylhydrazine
TXNIP	Thioredoxin-interacting protein
NLRP3	NOD-like receptor family pyrin domain-containing 3 inflammasome
PI3K/Akt	Phosphatidylinositol 3-kinase/protein kinase B pathway
ERK	Extracellular signal-regulated kinase
Hsp90α	Heat shock protein 90 alpha
AMPK	AMP-activated protein kinase
HIF-1α	Hypoxia-inducible factor 1 alpha
MMP-9	Matrix metalloproteinase-9
TNF-α	Tumor necrosis factor alpha
VEGF	Vascular endothelial growth factor
BSA	Bovine serum albumin
SEM	Standard error of the mean
ns	Not significant
